# BRAF/MEK inhibitors promote CD47 expression that is reversible by ERK inhibition in melanoma

**DOI:** 10.18632/oncotarget.17704

**Published:** 2017-05-09

**Authors:** Fen Liu, Chen Chen Jiang, Xu Guang Yan, Hsin-Yi Tseng, Chun Yan Wang, Yuan Yuan Zhang, Hamed Yari, Ting La, Margaret Farrelly, Su Tang Guo, Rick F. Thorne, Lei Jin, Qi Wang, Xu Dong Zhang

**Affiliations:** ^1^ Department of Respiratory Medicine, The Second Hospital, Dalian Medical University, Dalian, China; ^2^ School of Medicine and Public Health, The University of Newcastle, NSW, Australia; ^3^ School of Biomedical Sciences and Pharmacy, The University of Newcastle, NSW, Australia; ^4^ School of Environmental and Life Sciences, University of Newcastle, NSW, Australia

**Keywords:** CD47, BRAF/MEK inhibitors, NRF-1, melanoma, phagocytosis

## Abstract

The expression of CD47 on the cancer cell surface transmits “don’t eat me” signalling that not only inhibits phagocytosis of cancer cells by phagocytes but also impairs anti-cancer T cell responses. Here we report that oncogenic activation of ERK plays an important role in transcriptional activation of CD47 through nuclear respiratory factor 1 (NRF-1) in melanoma cells. Treatment with BRAF/MEK inhibitors upregulated CD47 in cultured melanoma cells and fresh melanoma isolates. Similarly, melanoma cells selected for resistance to the BRAF inhibitor vemurafenib expressed higher levels of CD47. The increase in CD47 expression was mediated by ERK signalling, as it was associated with rebound activation of ERK and co-knockdown of ERK1/2 by siRNA diminished upregulation of CD47 in melanoma cells after exposure to BRAF/MEK inhibitors. Furthermore, ERK1/2 knockdown also reduced the constitutive expression of CD47 in melanoma cells. We identified a DNA fragment that was enriched with the consensus binding sites for NRF-1 and was transcriptionally responsive to BRAF/MEK inhibitor treatment. Knockdown of NRF-1 inhibited the increase in CD47, indicating that NRF-1 has a critical role in transcriptional activation of CD47 by ERK signalling. Functional studies showed that melanoma cells resistant to vemurafenib were more susceptible to macrophage phagocytosis when CD47 was blocked. So these results suggest that NRF-1-mediated regulation of CD47 expression is a novel mechanism by which ERK signalling promotes the pathogenesis of melanoma, and that the combination of CD47 blockade and BRAF/MEK inhibitors may be a useful approach for improving their therapeutic efficacy.

## INTRODUCTION

Targeting BRAF, MEK, and co-targeting BRAF and MEK using specific inhibitors have become the standard of care for patients with late-stage mutant BRAF melanomas [1–3]. However, the benefits are often of limited duration due to rapid development of resistance [3]. Many mechanisms have been shown to contribute to the resistance [4–6]. However, the potential effect of BRAF/MEK inhibitors on the interaction between melanoma cells and the immune system seems paradoxical. On one hand, there is a large body of evidence showing that MAPK activation in melanoma cells contributes to immunosuppression and BRAF/MEK inhibitors trigger melanoma-specific immune responses, which are typically manifested by a rapid increase in tumour infiltrating lymphocytes (TILs) consisting mainly of cytotoxic T lymphocytes (CTLs) [7–12]. On the other, TIL numbers decline progressively after the initial increase, suggesting that the balance between immunostimulatory and immunosuppressive mechanisms in the melanoma environment skews from the former to the latter [7, 13]. While BRAF/MEK inhibitor-triggered anti-melanoma immune responses are associated with the increased expression of melanoma antigens and reduction in immunosuppressive cytokines such as interleukin (IL)-6 and IL-8 [10–12, 14], the immunosuppressive effect of the inhibitors has been linked to upregulation of programmed death ligand 1 (PD-L1) on the melanoma cell surface and stimulation of tumour-associated macrophages (TAMs) that in turn promote melanoma growth [9, 11, 13, 15].

Cluster of Differentiation 47 (CD47) is a transmembrane protein of the immunoglobulin superfamily that is ubiquitously expressed in mammalian tissues as a “self-marker” [16, 17]. It transmits “don’t eat me” signalling through interacting with signal-regulatory protein α (SIRPα) on the surface of phagocytic cells [16, 17]. Binding of SIRPα with CD47 results in phosphorylation of the immunoreceptor tyrosine-based inhibition motif (ITIM) on the cytoplasmic tail of SIRPα [16–18]. This results in inhibition of phagocytosis through preventing myosin-IIA accumulation at the phagocytic synapse [16–18]. In contrast to CD47, calreticulin (CRT), a protein normally located to the lumen of the endoplasmic reticulum (ER), transmits “eat-me” signalling once it is exposed to the outer leaflet of the cell membrane through binding to low density lipo-protein receptor–related protein 1 (LRP1) on the surface of phagocytic cell [16, 19]. Noticeably, CRT is often absent on the normal cell surface but is expressed on the surface of cancer cells and can be further induced when cells undergo immunogenic cell death [16–18].

The expression of CD47 is frequently increased in human cancer cells [17, 18, 20, 21]. Although the mechanism responsible for this remains largely unknown, blockade of the interaction between CD47 and SIRPα is emerging as a promising immunotherapeutic approach in the treatment of cancer [16, 18, 21–23]. Indeed, targeting CD47 has demonstrated potent preclinical activity against various cancers including melanoma [16, 18, 21–23]. Although this is directly related to phagocytosis of cancer cells by macrophages, CD47 blockade also triggers anti-cancer T cell responses through macrophages as well as dendritic cells (DCs) [23, 24]. Humanized anti-CD47 Abs have entered early clinical trials in the treatment of various types of cancers (clinicaltrials.gov).

To further understand the effect of BRAF/MEK inhibition on the interaction between melanoma cells and the immune system, we have examined the potential effect of BRAF/MEK inhibitors on the expression of CD47 in melanoma cells. We report here that treatment with BRAF or MEK inhibitors upregulates CD47 in melanoma cells *in vitro* and *in vivo*, and that melanoma cells resistant to BRAF inhibitors are more susceptible to macrophage phagocytosis upon CD47 blockade. Moreover, we show that the increase in CD47 expression triggered by BRAF and MEK inhibitors is mediated by the transcription factor nuclear respiratory factor 1 (NRF-1) and is reversible by ERK inhibition.

## RESULTS

### BRAF/MEK inhibitors upregulate CD47 expression in melanoma cells

**Figure 1:**
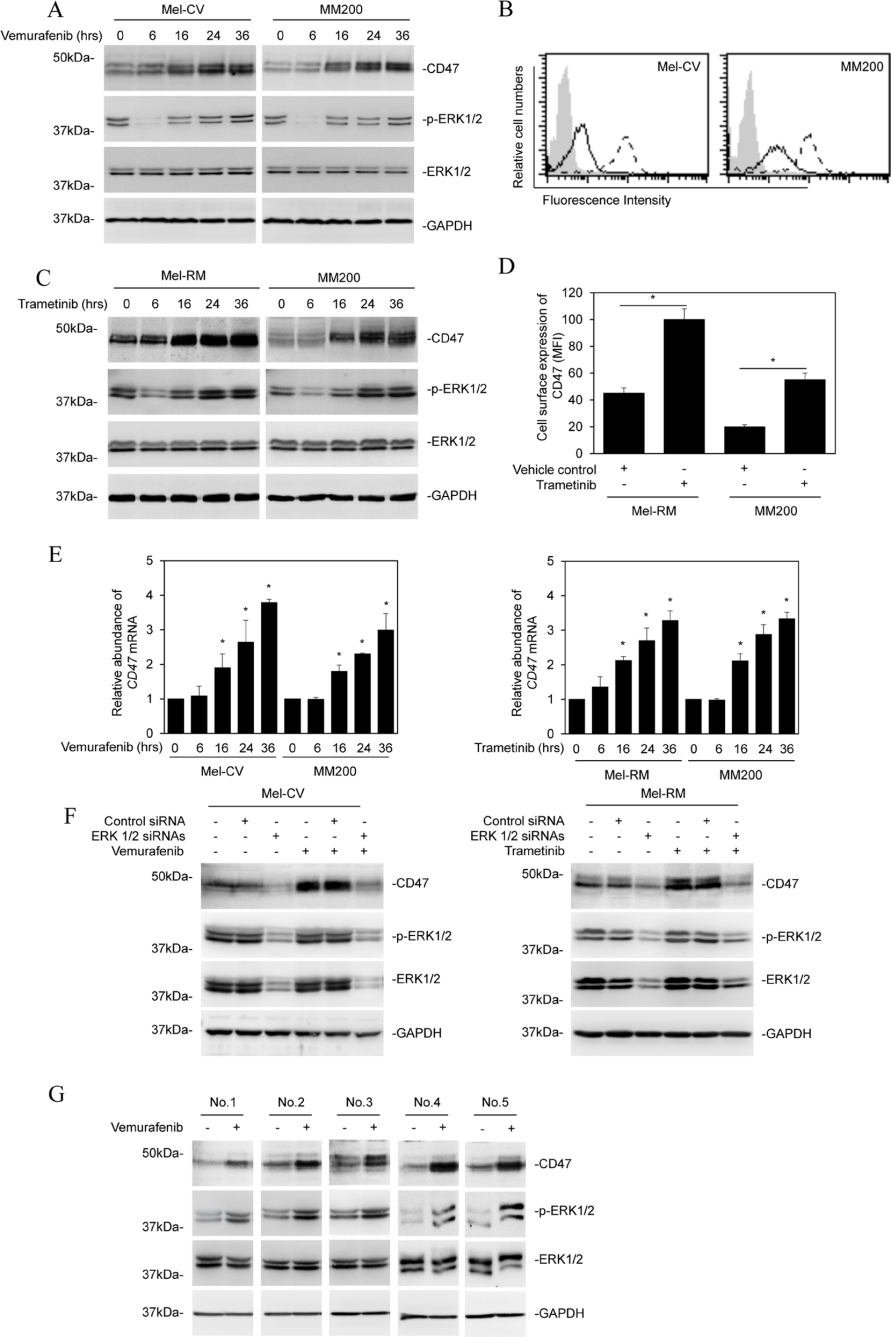
BRAF/MEK inhibitors upregulate CD47 in melanoma cells (**A**) Whole cell lysates from Mel-CV and MM200 cells treated with vemurafenib (3 μM) for indicated periods were subjected to Western blot analysis. Data shown are representative of three individual experiments. (**B**) Representative flow cytometry histograms showing that treatment with vemurafenib (3 μM) for 24 hours upregulated CD47 expression on the surface of Mel-CV and MM200 cells. Data shown are representative of three individual experiments. Filled histograms, isotype control; solid line histograms, before treatment; dotted line histograms, after treatment. (**C**) Whole cell lysates from Mel-RM and MM200 cells treated with trametinib (1 μM) for indicated periods were subjected to Western blot analysis. Data shown are representative of three individual experiments. (**D**) Comparison of the cell surface expression of CD47 presented as mean fluorescence intensity (MFI) of CD47 staining in Mel-RM and MM200 cells before and after treatment with trametinib (1 μM) for 24 hours (*n =* 3, mean ± S.E.M.; Student’s *t*-test, *P < 0.05). (**E**) Total RNA.s from Mel-CV and MM200 cells treated with vemurafenib (3 μM) (upper) and from Mel-RM and MM200 cells treated with trametinib (1 μM) (lower) for indicated periods were subjected to qPCR analysis. The relative abundance of CD47 mRNA in individual cell lines before treatment was arbitrarily designated as 1 (*n =* 3, mean ± S.E.M.; Student’s *t*-test, *P < 0.05). (**F**) Mel-CV (left) and Mel-RM (right) cells were transfected with the control or the combination of ERK1 and ERK2 siRNAs. Twenty-four hours later, Mel-CV and Mel-RM cells were respectively treated with vemurafenib (3 μM) and trametinib (1 μM) for a further 24 hours. Whole cell lysates were subjected to Western blot analysis. Data shown are representative of three individual experiments. (**G**) Whole cell lysates from the indicated fresh melanoma isolates treated with vemurafenib (3 μM) for 24 hours were subjected to Western blot analysis. Data shown are representative of three individual experiments.

We tested the potential effect of BRAF inhibitors on the expression of CD47 in melanoma cells by treating Mel-CV and MM200 cells (BRAF^V600E^) with the BRAF inhibitor vemurafenib for various periods. Vemurafenib upregulated CD47 expression in both Mel-CV and MM200 cells that was detectable at 16 hours with a further increase at 24 and 36 hours after treatment as shown by Western blot analysis of total protein extracts from whole cell lysates (Figure 1A). This increase in CD47 total protein expression was translated into upregulation of its expression on the cell surface as shown by flow cytometry analysis (Figure 1B). Similarly, treatment of MM200 cells and Mel-RM cells (wild-type BRAF) with the MEK inhibitor trametinib also resulted in upregulation of CD47 with comparable kinetics (Figure 1C and 1D). The increase in the CD47 protein expression triggered by vemurafenib or trametinib was associated with elevated mRNA expression (Figure 1E), which was due to a transcriptional increase rather than changes in its stability, as its turnover rates remained similar in cells before and after treatment with the inhibitors as shown in actinomycin D-chase assays (Supplementary Figure 1A).

Strikingly, the increase in CD47 coincided with rebound activation of ERK after treatment with vemurafenib or trametinib (Figure 1A and 1C) [25], suggesting that CD47 upregulation by these inhibitors may be associated with reactivation of ERK. Indeed, knockdown of ERK1/2 by siRNA diminished upregulation of CD47 by vemurafenib and trametinib (Figure 1F). Moreover, it markedly reduced the basal levels of CD47 expression (Figure 1F). The effect of BRAF/MEK inhibitors on the expression of CD47 was confirmed in additional two BRAF^V600E^ (IgR3 and Sk-Mel-28) and two wild-type BRAF (ME1007 and ME4405) melanoma cells lines treated with vemurafenib and trametinib, respectively (Supplementary Figure 1B). Furthermore, CD47 expression was upregulated by treatment with vemurafenib in a panel of fresh melanoma isolates carrying the BRAF^V600E^ mutation (Figure 1G) [25].Taken together, these results suggest that treatment with BRAF or MEK inhibitors upregulates CD47 expression due to reactivation of ERK.

### CD47 is upregulated in melanoma cells resistant to vemurafenib

**Figure 2:**
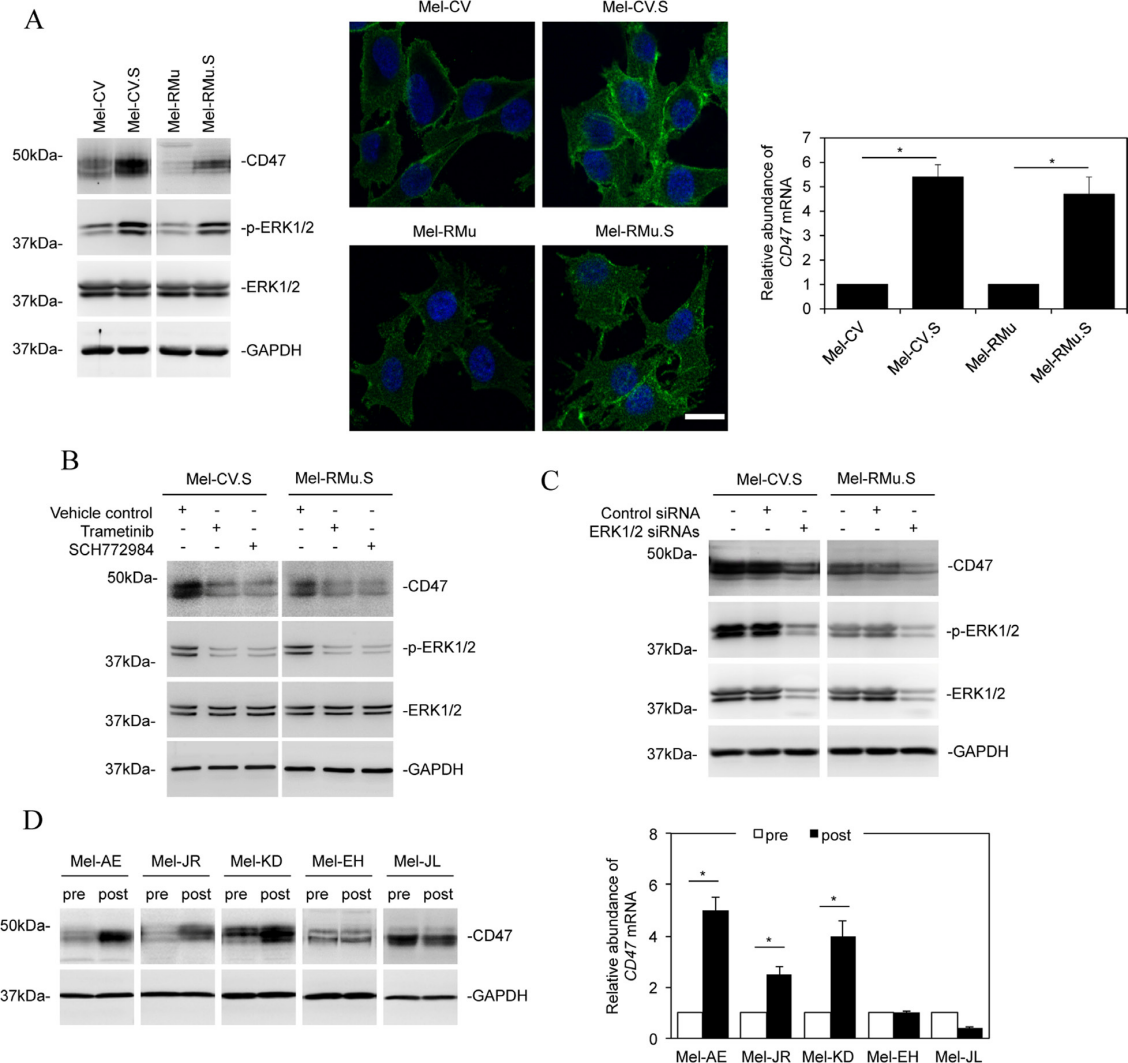
Melanoma cells resistant to vemurafenib express elevated levels of CD47 (**A**) Left: Whole cell lysates from Mel-CV, Mel-CV.S, Mel-RMu, and Mel-RMu.S cells were subjected to Western blot analysis. Data shown are representative of three individual experiments. Middle: cells of Mel-CV, Mel-CV.S, Mel-RMu, and Mel-RMu.S cells were subjected to immunofluorescence stainning. Right: Total RNAs from Mel-CV, Mel-CV.S, Mel-RMu, and Mel-RMu.S cells were subjected to qPCR analysis. The relative abundance of CD47 mRNA in individual parental cell lines was arbitrarily designated as 1 (*n =* 3, mean ± S.E.M.; Student’s *t*-test, *P < 0.05). (**B**) Whole cell lysates from Mel-CV.S and Mel-RMu.S cells treated with trametinib (1 μM) or SCH772984 (1 μM) were subjected to Western blot analysis. Data shown are representative of three individual experiments. (**C**) Mel-CV.S and Mel-RMu.S cells were transfected with the control or the combination of ERK1 and ERK2 siRNAs. Twenty-four hours later, whole cell lysates were subjected to Western blot analysis. Data shown are representative of three individual experiments. (**D**) Left: Whole cell lysates of the indicated paired pre- and post-treatment primary melanoma cultures were subjected to Western blot analysis. Data shown are representative of three individual experiments. Right: Total RNAs from the indicated paired pre- and post-treatment primary melanoma cultures were subjected to qPCR analysis. The relative abundance of CD47 mRNA in individual pre-treatment cultures was arbitrarily designated as 1 (*n =* 3, mean ± S.E.M.; Student’s *t*-test, *P < 0.05).

Reactivation of ERK is a major mechanism of acquired resistance of melanoma cells to BRAF inhibitors [3, 25]. We therefore examined CD47 expression in Mel-CV and Mel-RMu cells selected for resistance to vemurafenib by prolonged exposure to the inhibitor [25], which were respectively designated Mel-CV.S and Mel-RMu.S hereafter. As expected, the selected cells displayed higher levels of activated ERK1/2 than their corresponding parental counterparts (Figure 2A) [25], Along with this was the increased expression of CD47 at both the protein and mRNA levels (Figure 2A). Treatment of Mel-CV.S and Mel-RMu.S cells with trametinib or the ERK inhibitor SCH772984 inhibited ERK activation, which was associated with reduction in the expression of CD47 (Figure 2B), suggesting that upregulation of CD47 in vemurafenib-selected cells was mediated by activation of ERK. In support, siRNA knockdown of ERK1/2 reduced the expression of CD47 in Mel-CV.S and Mel-RMu.S cells (Figure 2C).

To examine the potential clinical relevance of CD47 upregulation in acquired resistance to BRAF inhibitors *in vivo*, we took advantage of primary melanoma cell cultures of paired BRAF^V600E^ melanoma biopsy samples from five patients pre- and post-treatment with vemurafenib [25]. These cultures represent cases where metastatic melanomas initially responded to vemurafenib but relapsed after various progression-free periods [25]. Analysis of both protein and mRNA levels showed that three of the five post-treatment primary cultures expressed significantly higher levels of CD47 than the corresponding pre-treatment cultures (Figure 2D). These findings provide evidence that systemic treatment with vemurafenib results in upregulation of CD47 in melanoma cells *in vivo*. Nonetheless, it has to be noted that CD47 upregulation may occur on a case-by-case basis (Figure 2D).

### Melanoma cells resistant to vemurafenib are more susceptible to macrophage phagocytosis upon CD47 blockade

**Figure 3:**
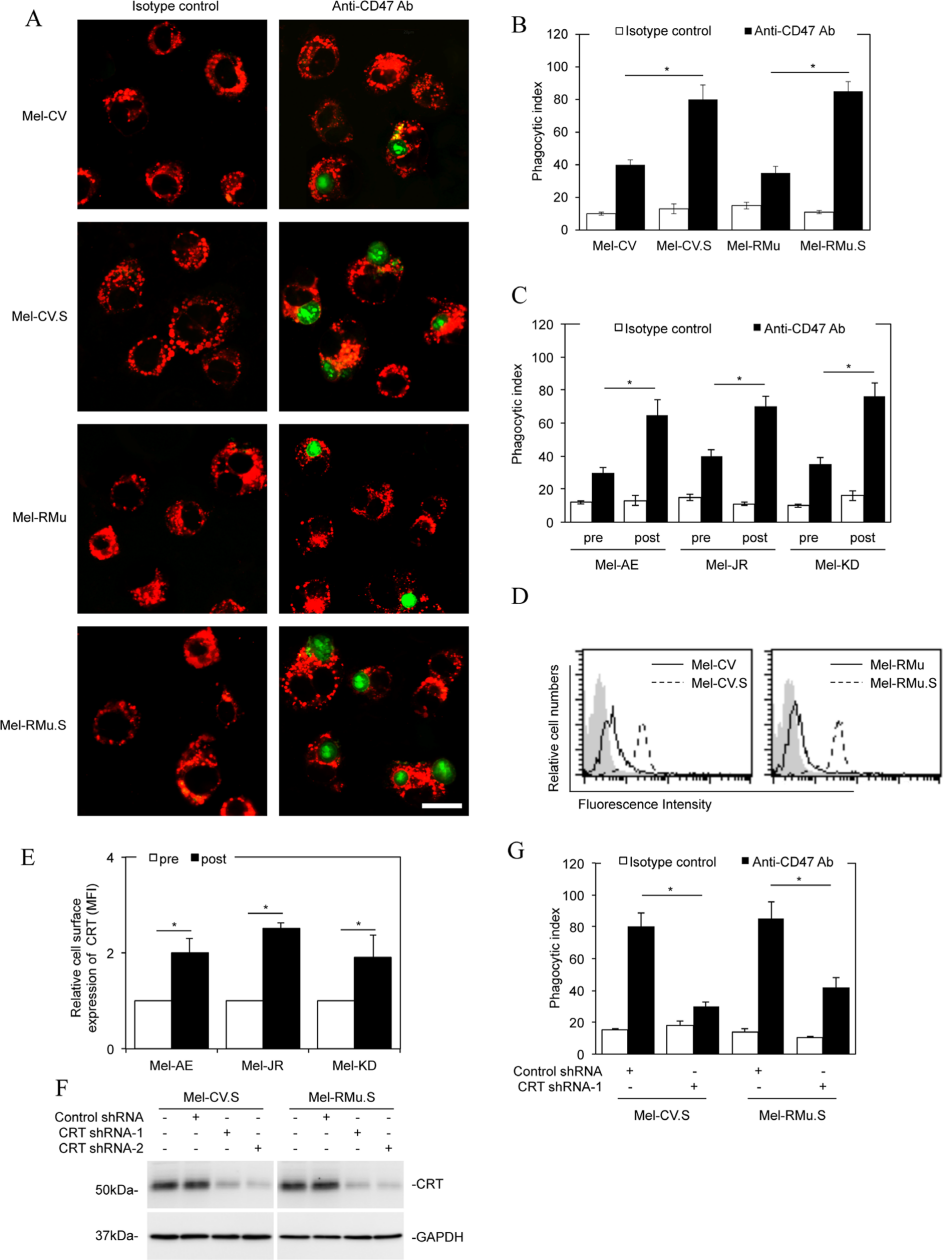
Melanoma cells resistant to vemurafenib are more susceptible to macrophage phagocytosis upon CD47 blockade (**A**) CFSE-labelled Mel-CV, Mel-CV.S, Mel-RMu, and Mel-RMu.S cells along with a blocking antibody against CD47 (B6H12.2, 10 μg/ml) were added into cultures of PKH26-labelled PBMC-derived macrophages. Two hours later, cultures were washed and examined with an inverted con-focal microscope. The data shown are representative microphotographs of three individual experiments. Green: CFSE-labelled melanoma cells; red: PKH26-labelled macrophages. Scale bar, 20 uM. (**B**) Comparison of the phagocytosis index of macrophage against melanoma cells of individual melanoma cell lines. The phagocytosis index was calculated as the number of phagocytized CFSE^+^ cells per 100 macrophages (*n =* 3, mean ± S.E.M.; Student’s *t*-test, *P < 0.05). (**C**) Comparison of the phagocytosis index of macrophage against melanoma cells of pre- and post-treatment primary cultures (*n =* 3, mean ± S.E.M.; Student’s *t*-test, *P < 0.05). (**D**) Representative flow cytometry histograms showing that Mel-CV.S and Mel-RMu.S expressed higher levels of CRT on the cell surface compared with Mel-CV and Mel-RMu cells, respectively. Data shown are representative of three individual experiments. Filled histograms, isotype control; solid line histograms, Mel-CV or Mel-RMu; dotted line histograms, Mel-CV.S or Mel-RMu.S. (**E**) Representative flow cytometry showing that CRT were expressed at higher levels on the surface of melanoma cells from post-treatment compared with pre-treatment primary cultures (*n =* 3, mean ± S.E.M.; Student’s *t*-test, *P < 0.05). (**F**) Whole cell lysates of Mel-CV.S and Mel-RMu.S cells transduced with the control or CRT shRNAs were subjected to Western blot analysis. Data shown are representative of three Western blot analyses. (**G**) Comparison of the macrophage phagocytosis index against Mel-CV.S and Mel-RMu.S with or without CRT knocked down (*n =* 3, mean ± S.E.M.; Student’s *t*-test, *P < 0.05).

We next examined the effect of the increased CD47 expression on macrophage phagocytosis of melanoma cells resistant to vemurafenib. To this end, we labelled Mel-CV.S and Mel-RMu.S and their parental counterparts with the green fluorescence dye carboxyfluorescein succinimidyl ester (CFSE) [21]. CFSE-labelled melanoma cells were added along with a blocking antibody against CD47 or the isotype control to cultures of human peripheral blood mononuclear cells (PBMC)-derived macrophages labelled with the red fluorescence dye PKH26 [26]. Strikingly, markedly more Mel-CV.S and Mel-RMu.S cells were phagocytized by macrophages than Mel-CV and Mel-RMu cells, respectively, when CD47 was blocked (Figure 3A and 3B). These results suggest that CD47 is more critically needed for protection of vemurafenib-resistant melanoma cells from phagocytosis by macrophages. In support, melanoma cells from post-treatment primary cultures with increased CD47 expression were more prone to macrophage phagocytosis than the corresponding pre-treatment cells upon CD47 blockade (Figure 3C). Moreover, treatment with vemurafenib for 36 hours resulted in enhanced macrophage phagocytosis of Mel-CV and MM200 cells when CD47 was blocked (Supplementary Figure 2).

In addition to “don’t eat me” signalling mediated by CD47, it was conceivable that susceptibility of melanoma cells to phagocytosis by macrophage was also influenced by altered “eat-me” signals transmitted by CRT. Noticeably, the cell surface expression of CRT was increased in Mel-CV.S and Mel-RMu.S cells compared with Mel-CV and Mel-RMu cells, respectively (Figure 3D). Similarly, CRT was also expressed at higher levels on the surface of melanoma cells from post-treatment compared with pre-treatment primary cultures (Figure 3E). These results suggest that enhanced macrophage phagocytosis of melanoma cells resistant to vemurafenib when CD47 is inhibited is associated with stronger phagocytic “eat-me” signals transmitted by the increased expression of CRT on the cell surface. Indeed, knockdown of CRT rescued Mel-CV.S and Mel-RMu.S cells from macrophage phagocytosis triggered by CD47 blockade (Figure 3F and 3G and Supplementary Figure 3A). Similar to vemurafenib-resistant melanoma cells, Mel-CV and MM200 cells treated with vemurafenib for 36 hours also displayed increased CRT on their surface (Supplementary Figure 3B).

### BRAF/MEK inhibitors activate the -272/-191 fragment of the CD47 promoter

**Figure 4:**
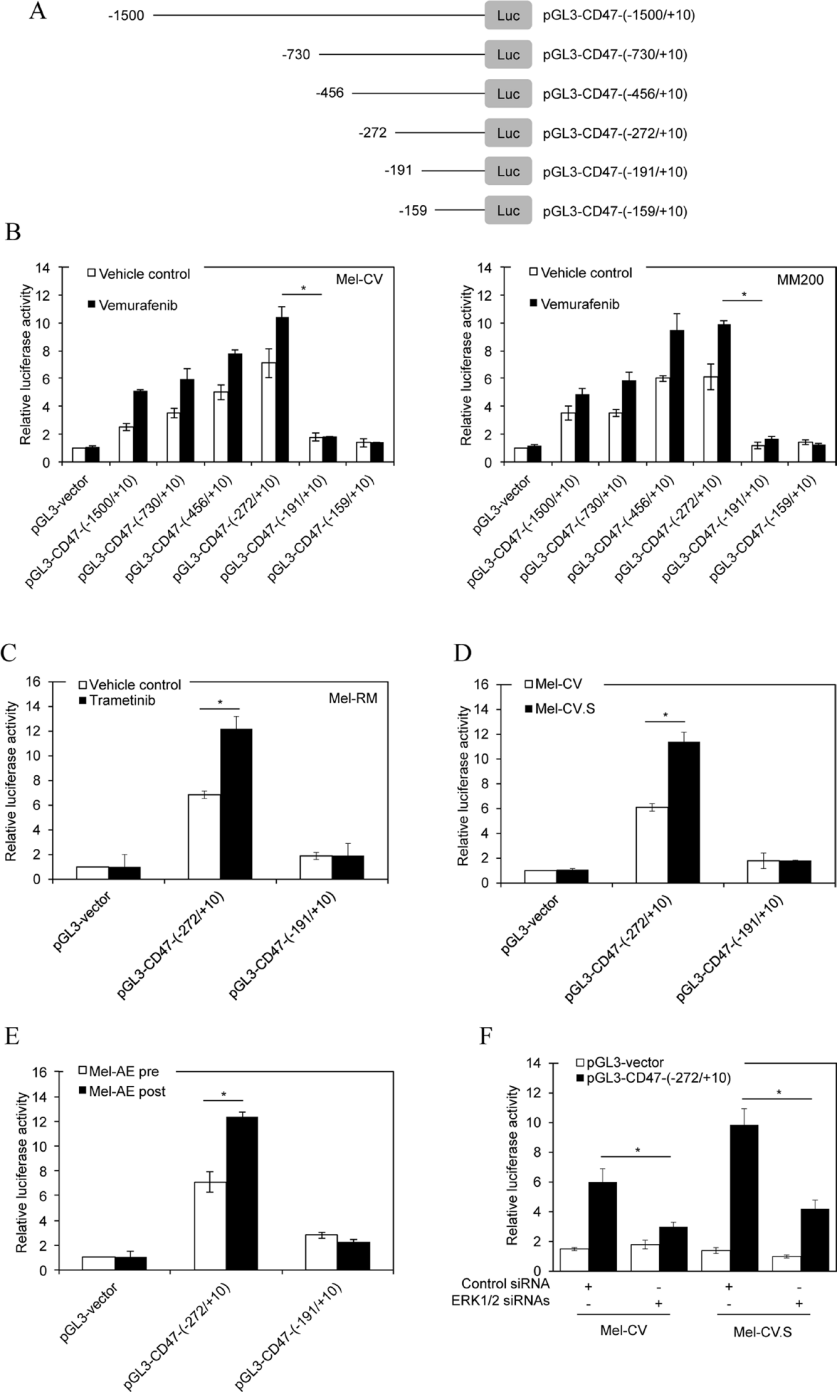
BRAF/MEK inhibitors activate the −**272/**−191 fragment of the CD47 promoter (**A**) A schematic illustration of construction of a series of incremental deletion luciferase reporter constructs. (**B**) Mel-CV (left) and MM200 (right) cells were transiently transfected with indicated pGL3 basic-based reporter constructs. Twenty-four hours later, cells were treated with vemurafenib (3 μM) for a further 16 hours followed by measurement of the luciferase activity (*n =* 3, mean ± S.E.M.; Student’s *t*-test, *P < 0.05). (**C**) Mel-RM cells were transiently transfected with indicated pGL3 basic-based reporter constructs. Twenty-four hours later, cells were treated with trametinib (1 μM) for a further 16 hours followed by measurement of the luciferase activity (*n =* 3, mean ± S.E.M.; Student’s *t*-test, *P < 0.05). (**D**) Mel-CV and Mel-CV.S cells were transiently transfected with indicated pGL3 basic-based reporter constructs. Twenty-four hours later, cells were subjected to measurement of the luciferase activity (*n =* 3, mean ± S.E.M.; Student’s *t*-test, *P < 0.05). (**E**) Cells of pre- and post-treatment Mel-AE primary cultures were transiently transfected with indicated pGL3 basic-based reporter constructs. Twenty-four hours later, cells were subjected to measurement of the luciferase activity (*n =* 3, mean ± S.E.M.; Student’s *t*-test, *P < 0.05). (**F**) Mel-CV and Mel-CV.S cells were co-transfected with the pGL3-CD47-(−272/+10) and the control or the combination of ERK1 and ERK2 siRNAs. Twenty-four hours later, cells were subjected to measurement of the luciferase activity (*n =* 3, mean ± S.E.M.; Student’s *t*-test, *P < 0.05).

Having demonstrated the functional significance of CD47 upregulation in protection of vemurafenib-resistant melanoma cells from macrophage phagocytosis, we focused on investigating transcriptional mechanisms responsible for vemurafenib-triggered upregulation of CD47 in melanoma cells. To determine the transcriptional region of the CD47 gene promoter that is responsive to vemurafenib, we introduced a series of pGL3 basic-based luciferase reporter constructs with incremental deletions from -1500bp upstream to 10bp downstream of the CD47 transcription start site into Mel-CV and Mel-RMu cells (Figure 4A) [27]. Treatment with vemurafenib markedly increased transcriptional activity in all the constructs except for pGL3-CD47-(−191/+10) and pGL3-CD47-(−159/+10) in Mel-CV and MM200 cells (Figure 4B). The shortest fragment that was transcriptionally responsive to vemurafenib was pGL3-CD47-(−272/+10) (Figure 4B). Therefore, the region between -272 and -191 is required for transcriptional upregulation of CD47 in melanoma cells by vemurafenib. In accordance, transcriptional activity of pGL3-CD47-(−272/+10) but not pGL3-CD47-(−191/+10) introduced into Mel-RM cells was increased by treatment with trametinib (Figure 4C). Moreover, pGL3-CD47-(−272/+10) but not pGL3-CD47-(−191/+10) displayed greater transcriptional activity in Mel-CV.S than Mel-CV cells, and in melanoma cells from a post-treatment primary culture with increased CD47 expression compared with those from the paired pre-treatment culture (Figure 4D and 4E). Transcriptional activation of the -272/-191 fragment of the CD47 promoter was dependent on ERK activity, as co-introduction of ERK1/2 siRNAs diminished transcriptional activity of pGL3-CD47-(−272/+10) in Mel-CV and Mel-CV.S cells (Figure 4F).

### NRF-1 is responsible for transcriptional upregulation of CD47 by BRAF/MEK inhibitors

**Figure 5:**
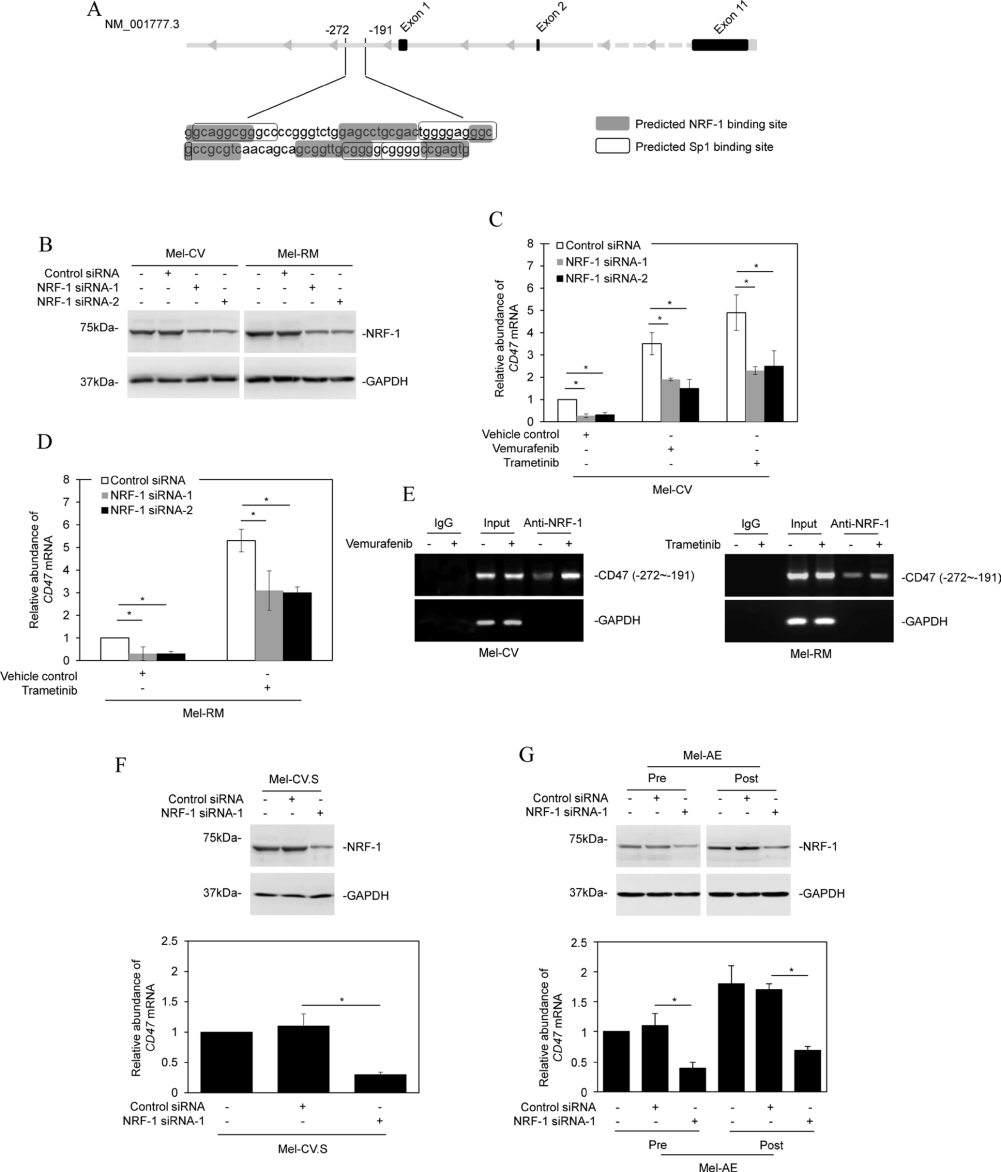
NRF-1 is responsible for transcriptional upregulation of CD47 by BRAF/MEK inhibitors (**A**) A schematic illustration of consensus binding sites for the transcription factors Sp1 and NRF-1 at the -272/-191 fragment of the CD47 promoter. (**B**) Mel-CV and Mel-RM cells were transfected with the control or NRF-1 siRNAs. Twenty-four hours later, whole cell lysates were subjected to Western blot analysis. Data shown are representative of three Western blot analyses. (**C**) Mel-CV cells were transfected with the control or NRF-1 siRNAs. Twenty-four hours later, cells were treated with vemurafenib (3 μM) or trametinib (1 μM) for a further 16 hours. Total RNAs were subjected to qPCR analysis of CD47 mRNA expression. The relative abundance of CD47 mRNA in cells transfected with the control siRNA was arbitrarily designated as 1 (*n =* 3, mean ± S.E.M.; Student’s *t*-test, *P < 0.05). (**D**) Mel-RM cells were transfected with the control or NRF-1 siRNAs. Twenty-four hours later, cells were treated trametinib (1 μM) for a further 16 hours. Total RNAs were subjected to qPCR analysis of CD47 mRNA expression. The relative abundance of CD47 mRNA in cells transfected with the control siRNA was arbitrarily designated as 1 (*n =* 3, mean ± S.E.M.; Student’s *t*-test, *P < 0.05). (**E**) Formaldehyde-cross-linked chromatin of Mel-CV cells with or without treatment with vemurafenib (3 μM) (left) and Mel-RM cells with or without treatment with trametinib (1 μM) (right) for 16 hours were subjected to immunoprecipitation with an antibody against NRF-1. The precipitates were subjected to PCR amplification using primers directed to the −272/−191 fragment of the CD47 promoter. Data shown are representative of three individual experiments. (**F**) Upper panel: Mel-CV.S cells were transfected with the control or NRF-1 siRNA (NRF-1 siRNA-1). Twenty-four hours later, whole cell lysates were subjected to Western blot analysis. Data shown are representative of three individual experiments. Lower panel: Mel-CV.S cells were transfected with the control or NRF-1 siRNA (NRF-1 siRNA-1). Twenty-four hours later, total RNAs were subjected to qPCR analysis of CD47 mRNA expression. The relative abundance of CD47 mRNA in cells transfected with the control siRNA was arbitrarily designated as 1 (*n =* 3, mean ± S.E.M.; Student’s *t*-test, *P < 0.05). (**G**) Upper panel: Cells of pre- and post-treatment Mel-AE primary cultures were transiently transfected with the control or NRF-1 siRNA (NRF-1 siRNA-1). Twenty-four hours later, whole cell lysates were subjected to Western blot analysis. Data shown are representative of three individual experiments. Lower panel: Cells of pre- and post-treatment Mel-AE primary cultures were transiently transfected with the control or NRF-1 siRNA (NRF-1 siRNA-1). Twenty-four hours later, total RNAs were subjected to qPCR analysis of CD47 mRNA expression. The relative abundance of CD47 mRNA in cells transfected with the control siRNA was arbitrarily designated as 1 (*n =* 3, mean ± S.E.M.; Student’s *t*-test, *P < 0.05).

The -272/-191 fragment of the CD47 promoter is enriched for consensus binding sites for the transcription factors Sp1 and NRF-1 (Figure 5A). However, only siRNA knockdown of NRF-1, but not knockdown of Sp1, abolished upregulation of CD47 mRNA by vemurafenib or trametinib in Mel-CV cells and by trametinib in Mel-RM cells, and reduced the basal levels of CD47 mRNA expression in the cells (Figure 5B–5D and Supplementary Figure 4A–4C). These results suggest that NRF-1 plays an important role in CD47 upregulation by BRAF or MEK inhibitors and may also be involved in regulating its constitutive expression in melanoma cells. In support, ChIP assays demonstrated that NRF-1 was associated with the -272/-191 fragment, and that this association was enhanced in Mel-CV cells treated with vemurafenib and in Mel-RM cells treated with trametinib (Figure 5E).

The role of NRF-1 in the expression of CD47 was confirmed by siRNA knockdown of NRF-1 in vemurafenib-resistant melanoma cells generated *in vitro* and *in vivo*, which showed that knockdown of NRF-1 reduced the expression levels of CD47 in Mel-CV.S cells and cells from a post-treatment primary culture with increased CD47 (Figure 5F and 5G). On the other hand, overexpression of NRF-1 reversed, at least in part, inhibition of CD47 expression by knockdown of ERK1/2 in MM200 and Mel-RM cells (Supplementary Figure 4D). Taken together, these results demonstrate that NRF-1 is responsible for BRAF/MEK-mediated regulation of CD47 expression in melanoma cells.

### ERK signalling regulates NRF-1 expression in melanoma cells

**Figure 6:**
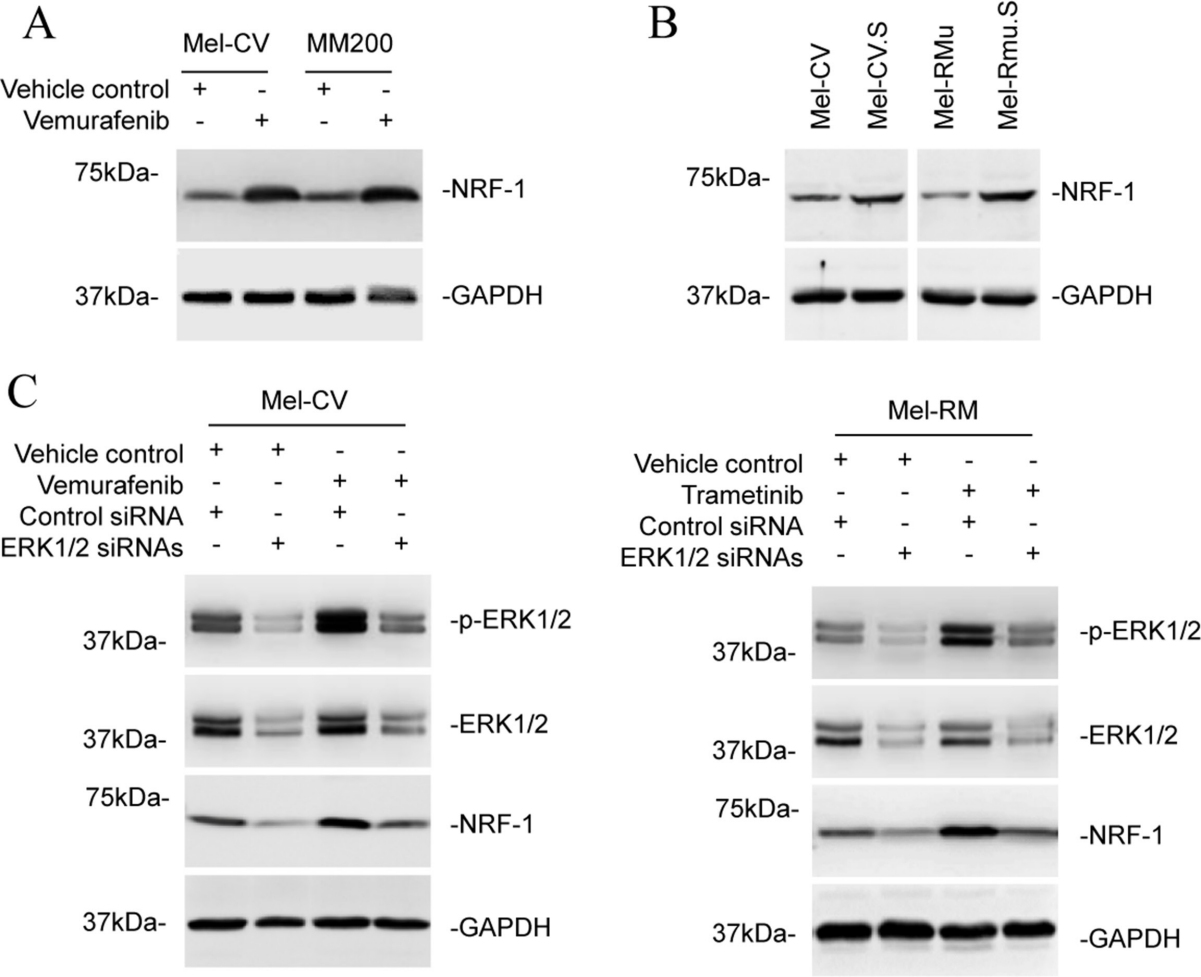
ERK signalling regulates NRF-1 expression in melanoma cells (**A**) Whole cell lysates from Mel-CV and MM200 cells with or without treatment with vemurafenib (3 μM) for 16 hours were subjected to Western blot analysis. Data shown are representative of three individual experiments. (**B**) Whole cell lysates from Mel-CV, Mel-CV.S, Mel-RMu, and Mel-RMu.S were subjected to Western blot analysis. Data shown are representative of three individual experiments. (**C**) Mel-CV (left) and Mel-RM (right) cells were transfected with the control or the combination of ERK1 and ERK2 siRNAs. Twenty-four hours later, Mel-CV and Mel-RM cells were treated with vemurafenib (3 μM) and trametinib (1 μM), respectively, for a further 24 hours. Whole cell lysates were subjected to Western blot analysis. Data shown are representative of three individual Western blot analyses.

We monitored the expression of NRF-1 in melanoma cells in response to treatment with BRAF and MEK inhibitors. Treatment of Mel-CV and MM200 cells with vemurafenib led to increases in NRF-1 expression (Figure 6A). Similarly, treatment of Mel-RM cells with trametinib also upregulated NRF-1 (Supplementary Figure 5A). Moreover, Mel-CV.S and Mel-RMu.S cells expressed elevated NRF-1 compared with Mel-CV and Mel-RMu cells, respectively (Figure 6B). These results suggest that reactivation of ERK after exposure to BRAF/MEK inhibitors may drive upregulation of NRF-1 in melanoma cells. This was confirmed by siRNA knockdown of ERK1/2, which inhibited upregulation of NRF-1 by vemurafenib in Mel-CV and by trametinib in Mel-RM cells (Figure 6C). Moreover, ERK1/2 knockdown reduced the expression of NRF-1 in Mel-CV.S cells, and in cells from a post-treatment primary culture (Supplementary Figure 5B). Therefore, ERK activation upregulates NRF-1 in melanoma cells.

## DISCUSSION

BRAF/MEK inhibitors have profound impacts on the interaction between melanoma cells and the immune system [7–15]. Overall, they trigger rapid melanoma-specific immune responses, which, however, succumb similarly in a fast fashion [7–10, 13]. The limited duration of the immune response conceivably contributes to the lack of long-term benefits of BRAF/MEK inhibitor treatment in the majority patients, as increasing evidence has shown that therapeutic drugs that lead to effective anti-cancer immune responses can achieve long-lasting tumour regression [28, 29]. A number of mechanisms such as the increased expression of programmed death ligand 1 (PD-L1) and exhaustion of T cells are involved in the relapse of anti-melanoma immune responses [9, 11, 13]. In addition, stimulation of TAMs by BRAF/MEK inhibitors also plays a role in promoting melanoma growth [15]. In this report, we present evidence that upregulation of CD47 expression is an important immunosuppressive mechanism triggered by BRAF/MEK inhibitors that prevents macrophage phagocytosis of melanoma cells, and may thus contribute to impairment of anti-melanoma T cell responses [23, 24].

As the only “don’t eat me” signal-generating protein on the target cell surface identified so far, CD47 expression is elevated on the surface of many types of cancer cells including melanoma cells [17–21]. Although the significance of the increase in CD47 expression in the pathogenesis of cancer remains to be fully elucidated, it is conceivable that the inhibitory effect of CD47 on phagocytosis of tumour cells by macrophages and DCs would impede processing and presentation of tumour antigens, and thus not only disable macrophage-mediated innate immune responses but also impair tumour-specific T cell responses against cancer [22, 23]. A number of mechanisms are emerging to be involved in upregulation of CD47 expression in cancer cells [30–32]. For example, hypoxia-inducible factor 1 (HIF-1) promotes CD47 expression in breast cancer cells under hypoxic conditions, whereas NF-κB mediates the increase in CD47 in hepatocellular carcinoma [30, 32]. Moreover, MYC-mediated regulation of CD47 plays an important role in the initiation and development of T cell acute lymphoblastic leukaemia [31]. It seems that mechanisms involved in regulation of CD47 expression may be highly cell type- and context-dependent. Our results showing that knockdown of ERK markedly inhibited the constitutive expression of CD47 indicate that its expression in melanoma cells is closely related to oncogenic activation of ERK. Whether ERK activation similarly plays a role in regulation of CD47 in other types of cancers remains to be investigated. It is known that there are considerable differences in oncogenic signalling context between melanoma and other cancers [33].

Paradoxically, treatment of melanoma cells or fresh melanoma isolates with BRAF/MEK inhibitors resulted in upregulation of CD47. This would argue against the role of the BRAF/MEK/ERK pathway in promoting CD47 expression in melanoma cells. Nevertheless, the increase in CD47 expression was associated with rebound activation of ERK and siRNA silencing of ERK1/2 diminished upregulation of CD47 by BRAF/MEK inhibitors, indicating CD47 upregulation after treatment with the inhibitors was due to resurgent ERK signalling. It remains puzzling that CD47 expression was not decreased when activation of ERK was reduced after treatment with BRAF/MEK inhibitors. A possible explanation for this is that the short-lived reduction in ERK activation caused by BRAF/MEK inhibitors was not adequate to manifest its impact on CD47 expression before ERK activation rebounded and drove signalling to upregulate CD47. Regardless, our results have clearly demonstrated the important role of reactivation of ERK in CD47 upregulation in melanoma cells by BRAF/MEK inhibitors. Moreover, activation of ERK was also important for the increased expression of CD47 in melanoma cells that acquired resistance to vemurafenib *in vitro* and *in vivo*. Of note, not all primary melanoma cell cultures of post-vemurafenib treatment biopsies expressed increased levels of CD47. This suggests that resistance to vemurafenib of melanomas without upregulation of CD47 may be primarily due to mechanisms other than reactivation of ERK, such as overexpression of MAP3K8 (COT) and switch of melanoma cells towards a more mesenchymal phenotype [34–36]. As a precedent, the expression of PD-L1 in melanoma cells is variably regulated by MAPK inhibitors [37, 38].

**Figure 7:**
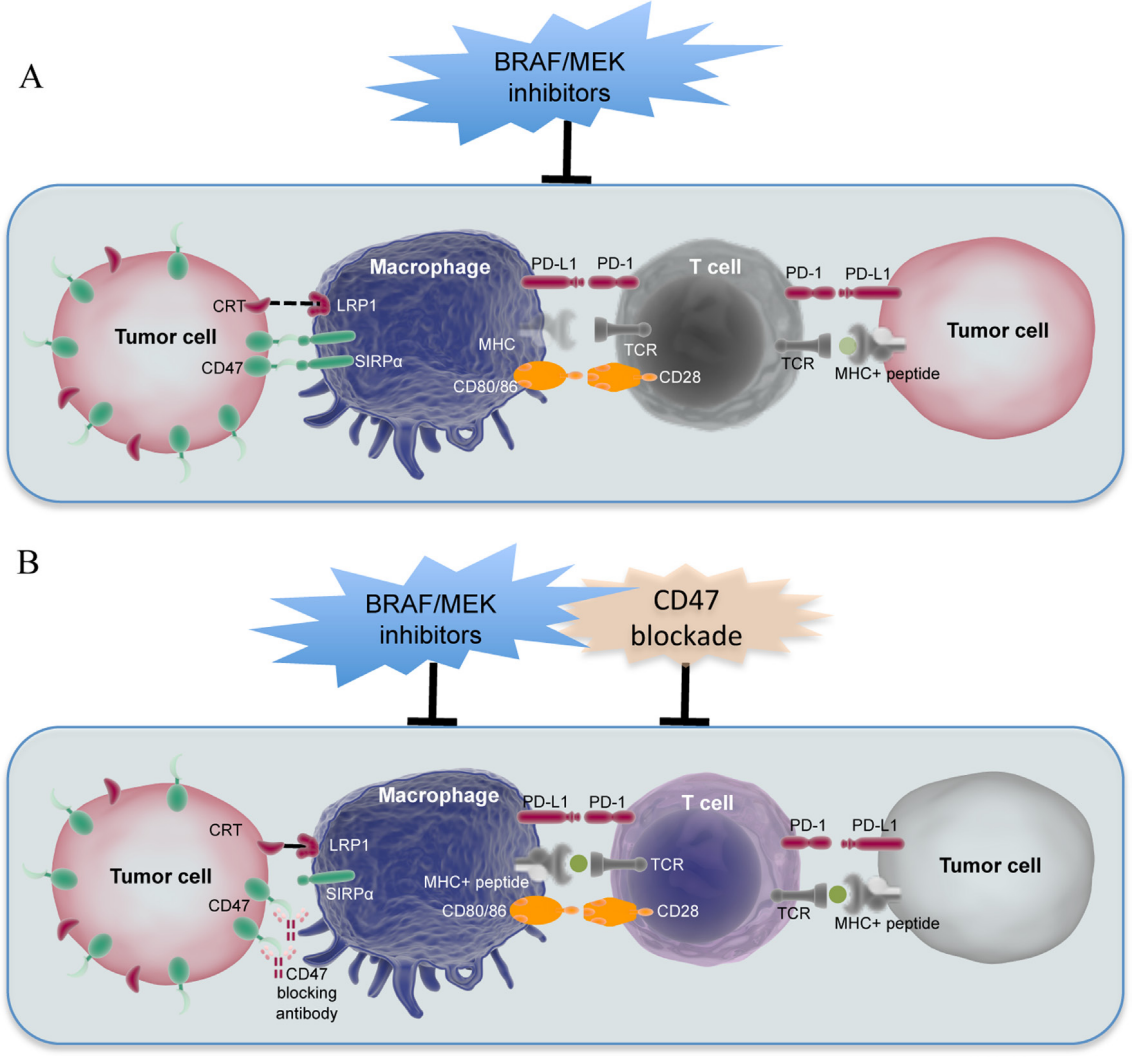
A proposed model in which CD47 upregulation regulates the interaction between melanoma cells and the immune system upon BRAF/MEK inhibitor treatment (**A**) Upregulation of CD47 in melanoma cells upon treatment with BRAF/MEK inhibitors blocks phagocytosis of melanoma cells by APCs, which in turn leads to compromised antigen processing and presentation to T cells. On the other hand, activated T cells may commit to apoptosis that presumably involves the increased interaction between PD-1 and PD-L1. (**B**) Blockade of CD47 in melanoma cells upon exposure to BRAF/MEK inhibitors enhances APC phagocytosis of melanoma cells that express elevated levels of CRT resulting from BRAF/MEK inhibitor treatment. Melanoma antigens are processed and presented to T cells, leading to T cell-mediated destruction of melanoma cells.

How does lymphocyte infiltration of the melanoma microenvironment decline after the initial increase by treatment with BRAF/MEK inhibitors remains to be fully understood. Our results suggest that blunting of the immune-response may be associated with the increase in CD47 expression in melanoma cells. Although the impact of CD47 on anti-cancer immune responses was originally attributed exclusively to its inhibitory effect on macrophage phagocytosis of cancer cells [16, 17, 21], it has been recently demonstrated that CD47 expression in cancer cells has a profound impact on anti-cancer T cell responses [23, 24, 39], as blockade of CD47 primes tumour-specific T cell responses as a consequence of enhanced phagocytosis of tumour cells by macrophages as well as DCs [23, 24, 39]. We therefore propose a model that the reduction in TIL numbers after the initial increase may involve the lack of antigen presentation to T cells by antigen presenting cells (APCs), which fail to capture antigens due to inhibition of their phagocytic activity by increased CD47 in melanoma cells (Figure 7). On the other hand, activated T cells in the melanoma microenvironment would perish through apoptosis that conceivably involves the increased interaction of PD-1 and PD-L1 (Figure 7) [40–42].

In support of the proposed model, melanoma cells resistant to vemurafenib appeared more susceptible to macrophage phagocytosis, suggesting that these cells are more critically dependent on CD47 for escaping the immune system. When the interaction of CD47 and SIRPα is blocked, a pro-phagocytic signal such as that transmitted by the CRT/ LRP1 system is needed for initiating phagocytosis [17, 18]. Indeed, vemurafenib-resistant melanoma cells expressed higher levels of CRT on the cell surface compared to their parental counterparts, which was responsible for the enhanced macrophage phagocytosis, as knockdown of CRT diminished phagocytosis of the cells by macrophages. It is known that induction of immunogenic cells death triggers exposure of CRT onto the cell surface [28, 29], but this is unlikely to play a role in the increased cell surface expression of CRT in vemurafenib-resistant melanoma cells, as these cells are viable and proliferative [43]. Irrespectively, these results demonstrate that high levels of CD47 are obliged for protection against macrophage phagocytosis of melanoma cells that express increased levels of CRT on the surface after exposure to BRAF/MEK inhibitors (Figure 7).

Another important finding of this study is that NRF-1 is responsible for ERK-mediated constitutive expression of CD47 and its upregulation upon BRAF/MEK inhibitor treatment in melanoma cells. This was evidenced by, 1) activation of ERK resulted in transcriptional activation of the -272/-191 fragment of the CD47 promoter; 2) this fragment is enriched of consensus binding sites for NRF-1; 3) NRF-1 binds to this fragment that was enhanced by BRAF/MEK inhibitors; 4) knockdown of NRF-1 abolished ERK-mediated upregulation of CD47; In addition, the role of NRF-1 in ERK-mediated CD47 upregulation is supported by the finding that ERK signalling promotes NRF-1 expression. The physiological role of NRF-1 is regulation of transcription of nuclear-encoded mitochondrial genes and thus mediates the biogenomic coordination between nuclear and mitochondrial genomes [44, 45]. Its activation involves phosphorylation and relocation from the cytoplasm to the nucleus [44, 46]. Whether ERK signalling causes these changes in NRF-1 in melanoma cells remains to be determined. ERK has been reported to mediate activation of NRF-1 by low-dose radiation in human skin fibroblast cells [46]. Little is known about the role of NRF-1 in the pathogenesis of cancer except that it promotes survival and proliferation of breast cancer cells [47, 48]. Our results now suggest that NRF-1 may play a part in protection of melanoma cells from the immune system through CD47. In support, NRF-1 regulates CD47 expression in human neuroblastoma and hepatoma cells [49].

## MATERIALS AND METHODS

### Cell culture and human tissues

The human melanoma cell lines were described previously [25]. Human fresh melanoma isolates were prepared as described previously [25]. Cancer cell line authentication was confirmed every 6 months using the AmpFISTR Identifiler PCR Amplification Kit from Applied Biosystems (Mulgrave, VIC, Australia) and GeneMarker V1.91 software (SoftGenetics LLC, State College, PA, USA). Resulting cell line STR profiles were cross-compared, where available, with the ATCC’s and German Collection of Microorganisms and Cell cultures’ online databases (Braunschweig, Germany) [25]. Cell lines were regularly tested for mycoplasma infection using Myco Alert according to the manufacturer’s protocol (Lonza, Walkersville, MD, USA). Studies using human tissues were approved by the Human Research Ethics Committees of the University of Newcastle.

### Antibodies and reagents

Antibodies (Abs), reagents and sequences of siRNAs are listed in Supplementary Tables 1–3.

### Phagocytosis assay

Human peripheral blood mononuclear cells (PBMC) were isolated from whole blood donated by healthy volunteers using Ficoll HyPaque (GE healthcare) and re-suspended in RPMI-1640 supplemented with 10% (v/v) FCS, 100 U/ml penicillin, 100 μg/ml streptomycin, 2 mM L-glutamine. Isolated PBMCs were seeded on 24-well plates for 2 hours at 37°C, 5% (v/v) CO_2_ to allow monocytes to adhere to the plate. Non-adherent cells were aspirated and monocytes were incubated with fresh complete media containing M-CSF (100 ng/ml; PeproTech). Monocytes were incubated for further 12 days to allow full differentiation into macrophages. Fresh media containing M-CSF were replenished on day 4 and 7. On the day performing phagocytosis assay, melanoma cells were fluorescently labelled with carboxyfluorescein succinimidyl ester (CFSE) (Invitrogen) and macrophages were labelled with PKH26 (Sigma-Aldrich) according to the manufacturer’s protocol. 2 × 10^5^ CFSE^+^ melanoma cells were incubated with 10ug/ml CD47 blocking antibody (B6H12.2) or isotype control in 1ml serum free RPMI media at 37°C, 5% (v/v) CO_2_ with an end-to-end rotating for 30 minutes. Melanoma cells were then co-cultured with macrophages, which were pre-incubated with serum free RPMI media for 2 hours. The mixed culture plates were then spun at 1000 rpm for 1 minute, and incubated at 37°C for two hours. Tumour cells that were not phagocytized were gently washed away using PBS and macrophages were fixed with 1% formaldehyde for 10 minutes and imaged with FV10i LIV confocal microscope (Olympus Australia Pty Ltd, Notting Hill, VIC Australia). The phagocytic index was calculated as the number of phagocytized CFSE^+^ cells per 100 macrophages [21, 22].

### Short hairpin RNA (shRNA)

shRNA was carried out as described before [25]. In brief, human shRNA lentiviral transduction particles against. CRT (SHCLNV-NM_004343 and sc-29234-V) as well as the corresponding control particles were purchased from Sigma-Aldrich (Castle Hill, NSW, Australia) or Santa Cruz (Santa Cruz, CA), respectively. shRNAs were used to infect cells according to the manufacturer’s protocol.

### Lentiviral gene transduction and DNA constructs

The *NRF-1* cDNA was cloned into the lentiviral expression plasmid pCDH-CMV-MCS-EF1-copGFP (Integrated Sciences, Chatswood, NSW, Australia). Transduction efficiency was monitored by detecting GFP via flow cytometry.

### Quantitative reverse transcription-PCR (qPCR)

qPCR was carried out as described before [25]. The primer sequences are: CD47, forward, 5′-CCTTCGTCATTGCCATATTG-3′, reverse, 5′-TAGGAGGTTGTATAGTCTTCTG-3′; CRT, forward, 5′- GCACTTGGATCCACCCAGAA-3′, reverse, 5′- ATGGTGCCAGACTTGACCTG-3′; β-actin, forward, 5′-GGCACCCAGCACAATGAAG-3′, reverse, 5′-GCCGATCCACACGGAGTAC T-3′. The relative expression level of CD47 or CRT mRNA was normalized against β-actin mRNA.

### Chromatin immunoprecipitation (ChIP) assays

ChIP assays were performed using the MAGnify™ Chromatin Immunoprecipitation System (Life Technologies, Scoresby, VIC, Australia) according to manufacturer’s instructions and as described previously [25]. In brief, cells were cross-linked with 1% formaldehyde. The bound DNA fragments were subjected to PCR reactions using the primer pairs: CD47 (-272∼-191), forward, 5′-GACAGGACGTGACCTGGA-3′, reverse, 5′-ACAGGCAGGACCCACTG-3′; GAPDH, forward, 5′-TACTAGCGGTTTTACGGGCG-3′, reverse, 5′-TCGAACAGGAGGAGCAGAGAGCGA-3′.

### Luciferase reporter assays

The fragment of the CD47 regulatory regions was cloned by PCR using human genomic DNA as a template. The fragment was then cloned into the luciferase reporter plasmid pGL3-Basic Luciferase Vector (Promega). Cells were transiently transfected with desired pGL3 basic-based constructs. Luciferase activity was measured using the Dual-Glo^®^ Luciferase Assay kit (Promega) with a Synergy 2 multi-detection microplate reader (BioTek, VT).

### Statistical analysis and data presentation

Statistical analysis was performed using JMP Statistics Made Visual™ software. Student’s *t*-test was used to assess differences between different groups. A *P* value less than 0.05 was considered statistically significant.

## CONCLUSIONS

In summary, we have shown in this study that treatment with BRAF/MEK inhibitors upregulates CD47 expression through NRF-1, and that this is mediated by reactivation of ERK in melanoma cells. Moreover, we demonstrate that the constitutive expression of CD47 in melanoma cells is also associated with ERK signalling. The functional significance of upregulation of CD47 by BRAF/MEK inhibitors is revealed by increased susceptibility of melanoma cells resistant to BRAF inhibitors to macrophage phagocytosis when CD47 is inhibited. The clinical relevance of these findings is supported by studies in fresh melanoma isolates and paired primary cultures established from melanoma biopsies of patients pre- and post-treatment with vemurafenib. Collectively, these results indicate that CD47 upregulation may be an important mechanism that stifles the anti-melanoma immune response initially activated by BARF/MEK inhibitors, and suggest that the combination of CD47 blockade and BRAF/MEK inhibitors simultaneously or sequentially may be a promising approach to improve their therapeutic efficacy. Of note, it has been recently reported that administration of chemotherapy before, but not after, CD47 blocking antibodies resulted in synergistic tumour control in a mouse lymphoma model, as explained in the paper, chemotherapy may synergize with anti-CD47 by increasing release of antigens and DNA from dying tumor cells; contrarily, chemotherapy administered after anti-CD47 therapy had detrimental effects on development of beneficial antitumor memory immune responses [23]. According to the result of our *in vitro* phagocytosis assay, melanoma cells resistant to vemurafenib were more susceptible to macrophage phagocytosis when CD47 was blocked. We propose that tumor cells in melanoma patients that have gained resistance to BRAF/MEK inhibitors may display the same feature. These results suggest that the combination of BRAF/MEK inhibitors and CD47 blocking antibodies may have synergistic effect by promoting macrophages phagocytosis of melanoma cells and may further trigger specific immune specific immunity response by presenting processed tumor cell antigens to T cells.

### Ethics approval and consent to participate

The research protocol was reviewed and approved by the Ethical Committee and Institutional Review Board of the Newcastle University. All human tissue samples were obtained with written informed consent from all subjects. All samples were anonymous.

## SUPPLEMENTARY MATERIALS FIGURES AND TABLE



## Abbreviations

ChIP: Chromatin immunoprecipitation
NRF-1: nuclear respiratory factor 1
TILs: tumour infiltrating lymphocytes
CTLs: cytotoxic T lymphocytes
PD-L1: programmed death ligand 1
TAMs: tumour-associated macrophages
CD47: cluster of differentiation 47
SIRPα: signal-regulatory protein α
ITIM: immunoreceptor tyrosine-based inhibition motif
CRT: calreticulin
ER: endoplasmic reticulum
LRP1: lipo-protein receptor-related protein 1
DCs: dendritic cells
CSFE: carboxyfluorescein succinimidy1 ester
PBMC: peripheral blood mononuclear cells
APCs: antigen presenting cells
