# Supplementary material for: BRAF/MEK inhibitors promote CD47 expression that is reversible by ERK inhibition in melanoma
Source: Oncotarget. 2017 May 9;8(41):69477–92. doi: 10.18632/oncotarget.17704 (PMC5642493; doi:10.18632/oncotarget.17704)
Supplement: Supplementary file 1 [file oncotarget-08-69477-s001.pdf]

BRAF/MEK inhibitors promote CD47 expression that is reversible by ERK inhibition in melanoma

Supplementary Materials

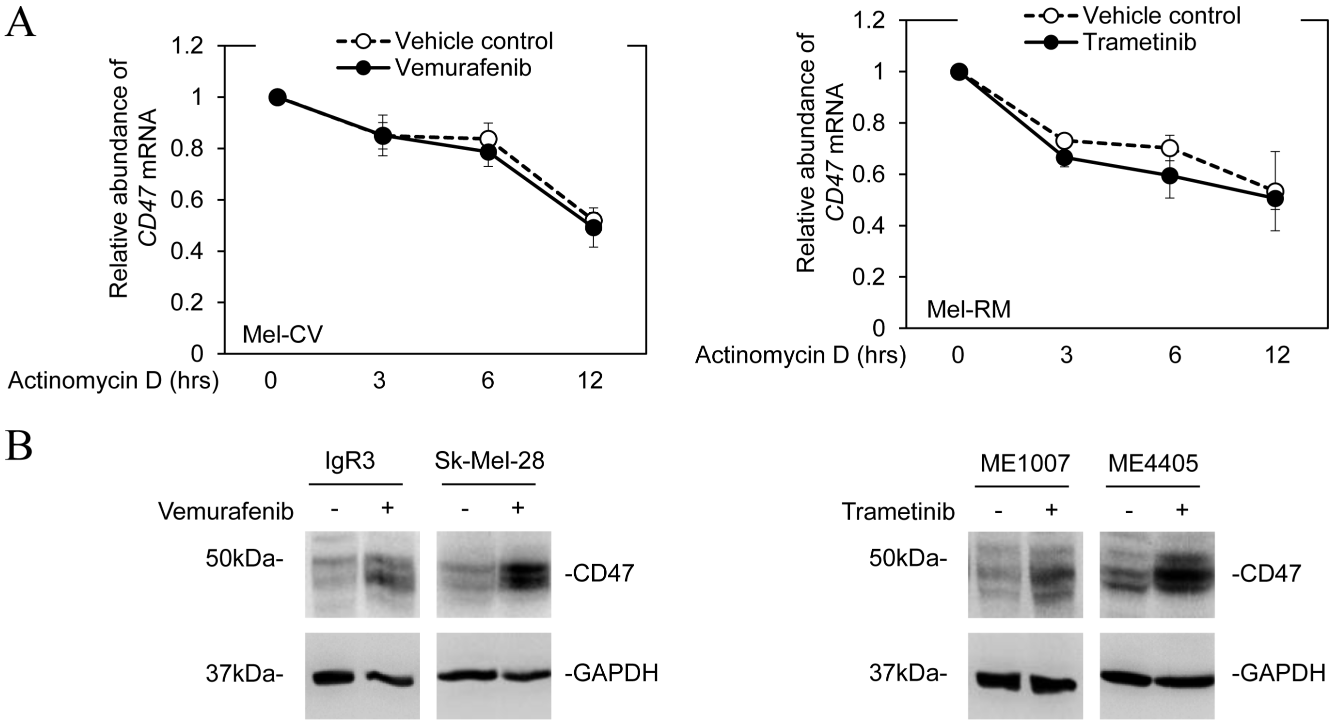

Supplementary Figure 1: (**A**) Total RNAs from Mel-CV cells treated with vemurafenib (3 μM) and Mel-RM cells treated with trametinib (1 μM) for indicated periods with or without pretreatment with actinomycin D (10 μg/ml) were subjected to qPCR analysis. The relative abundance of CD47 mRNA in individual cell lines before treatment was arbitrarily designated as 1 ($n = 3$, mean ± S.E.M.). (**B**) Whole cell lysates from the indicated melanoma cell lines with or without treatment with vemurafenib (3 μM) or trametinib (1 μM) for 24 hours were subjected to Western blot analysis. Data shown are representative of three individual experiments.

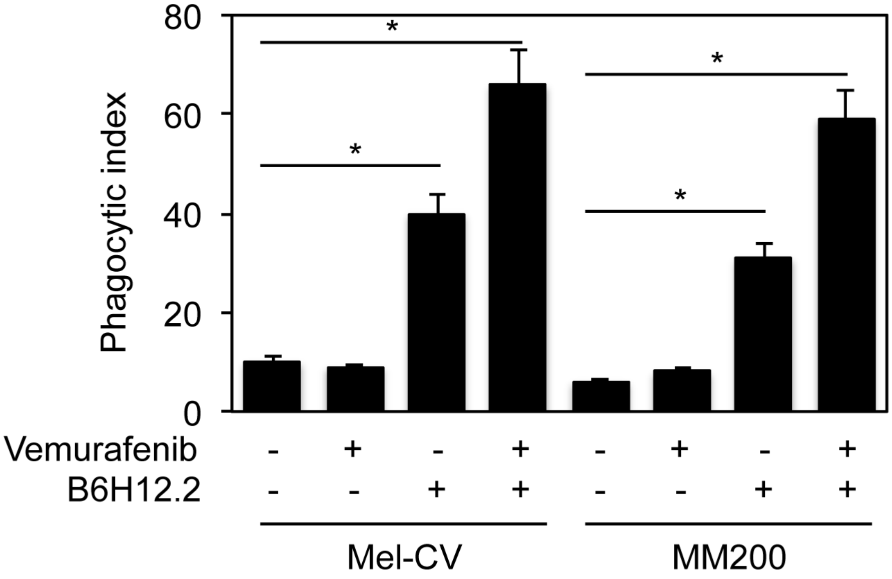

Supplementary Figure 2: CFSE-labelled Mel-CV and MM200 cells treated with vemurafenib (3 µM) for 36 hours were added along with a blocking antibody against CD47 (B6H12.2, 10 µg/ml) to cultures of macrophages that were labelled with PKH26. Two hours later, non-adherent cells were washed away, and the cultures were examined using a fluorescence microscope. The phagocytosis index was calculated as the number of phagocytized CFSE$^+$ cells per 100 macrophages ($n = 3$, mean ± S.E.M.; Student's t-test, $^*P < 0.05$).

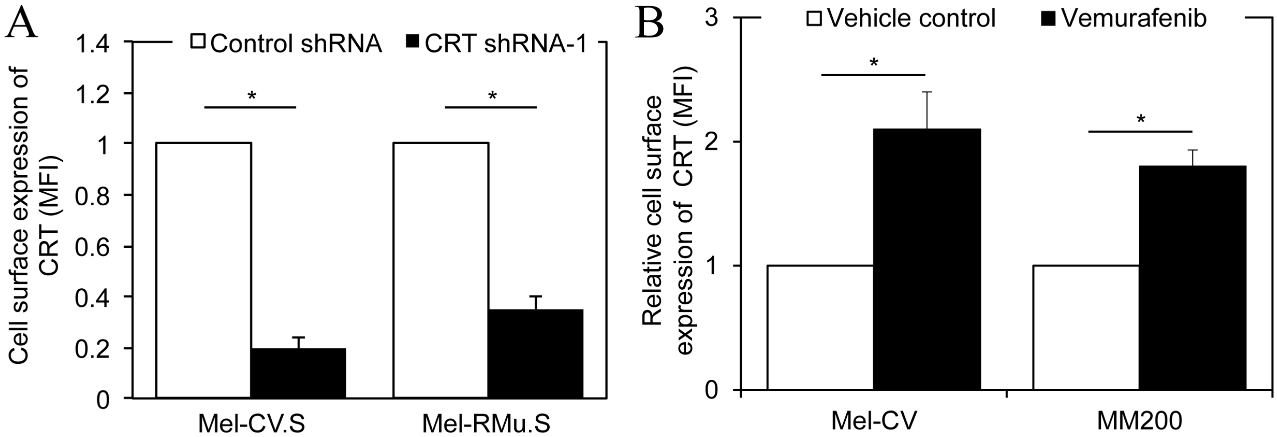

Supplementary Figure 3: (**A**) Comparison of the cell surface expression of CRT presented as mean fluorescence intensity (MFI) of CRT staining in Mel-CV.S and Mel-RMu.S cells transduced with the control and CRT shRNA-1 ($n = 3$, mean ± S.E.M.; Student's t-test, $^*P < 0.05$). (**B**) Comparison of the cell surface expression of CRT presented as mean fluorescence intensity (MFI) of CRT staining in Mel-CV and MM200 cells with or without treatment with vemurafenib (3 µM) for 36 hours ($n = 3$, mean ± S.E.M.; Student's t-test, $^*P < 0.05$).

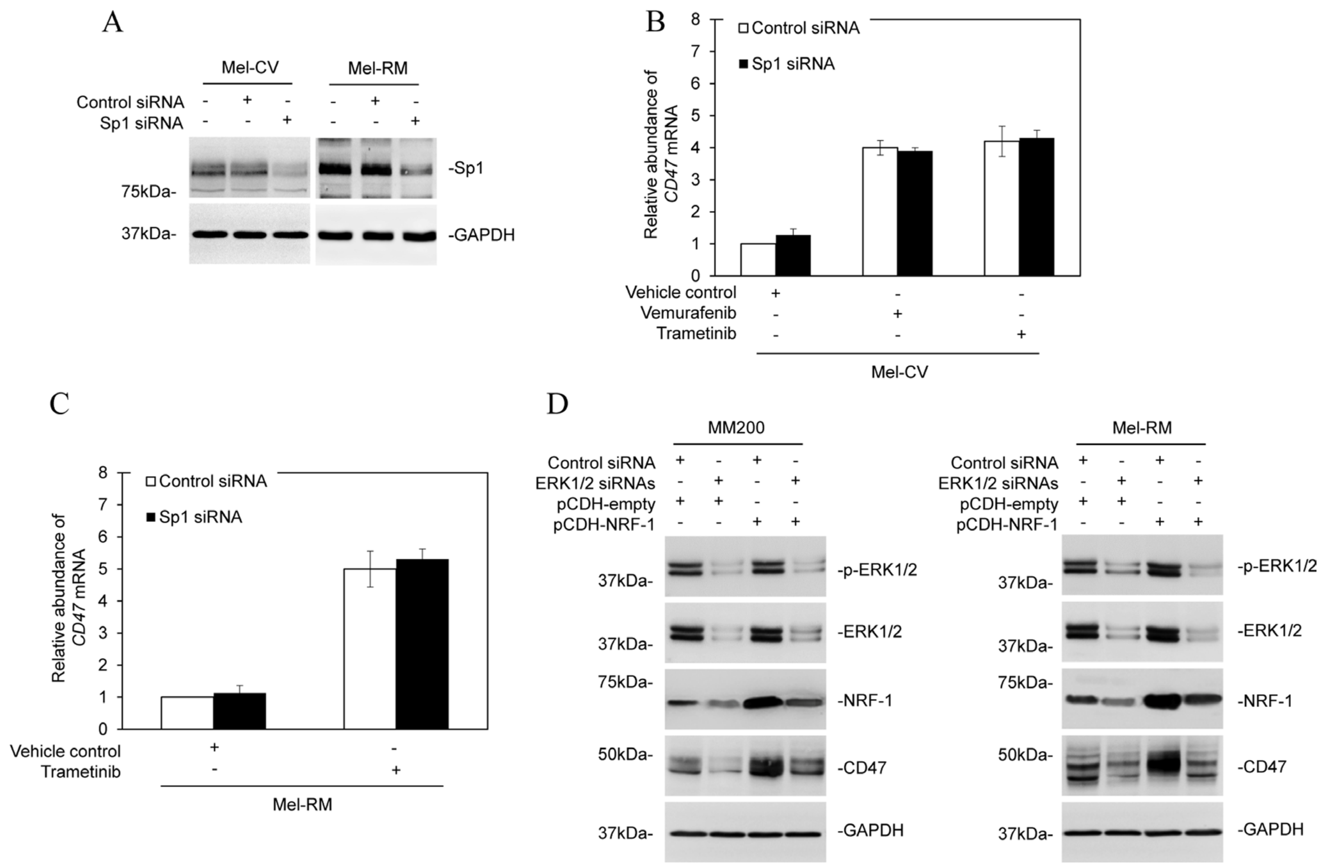

Supplementary Figure 4: (**A**) Mel-CV and Mel-RM cells were transfected with the control or Sp1 siRNA. Twenty-four hours later, whole cell lysates were subjected to Western blot analysis. Data shown are representative of three Western blot analyses. (**B**) Mel-CV cells were transfected with the control or Sp1 siRNA. Twenty-four hours later, cells were treated with vemurafenib (3 μM) or trametinib (1 μM) for a further 16 hours. Total RNAs were subjected to qPCR analysis of CD47 mRNA expression. The relative abundance of CD47 mRNA in cells transfected with the control siRNA was arbitrarily designated as 1 (n = 3, mean ± S.E.M.). (**C**) Mel-RM cells were transfected with the control or Sp1 siRNA. Twenty-four hours later, cells were treated trametinib (1 μM) for a further 16 hours. Total RNAs were subjected to qPCR analysis of CD47 mRNA expression. The relative abundance of CD47 mRNA in cells transfected with the control siRNA was arbitrarily designated as 1 (n = 3, mean ± S.E.M.). (**D**) MM200 (left) and Mel-RM (right) cells stably overexpressing NRF-1 were introduced with the control siRNA or the combination of ERK1 and ERK2 siRNAs. Twenty-four hours later, whole cell lysates were subjected to Western blot analysis. Data shown are representative of three individual experiments.

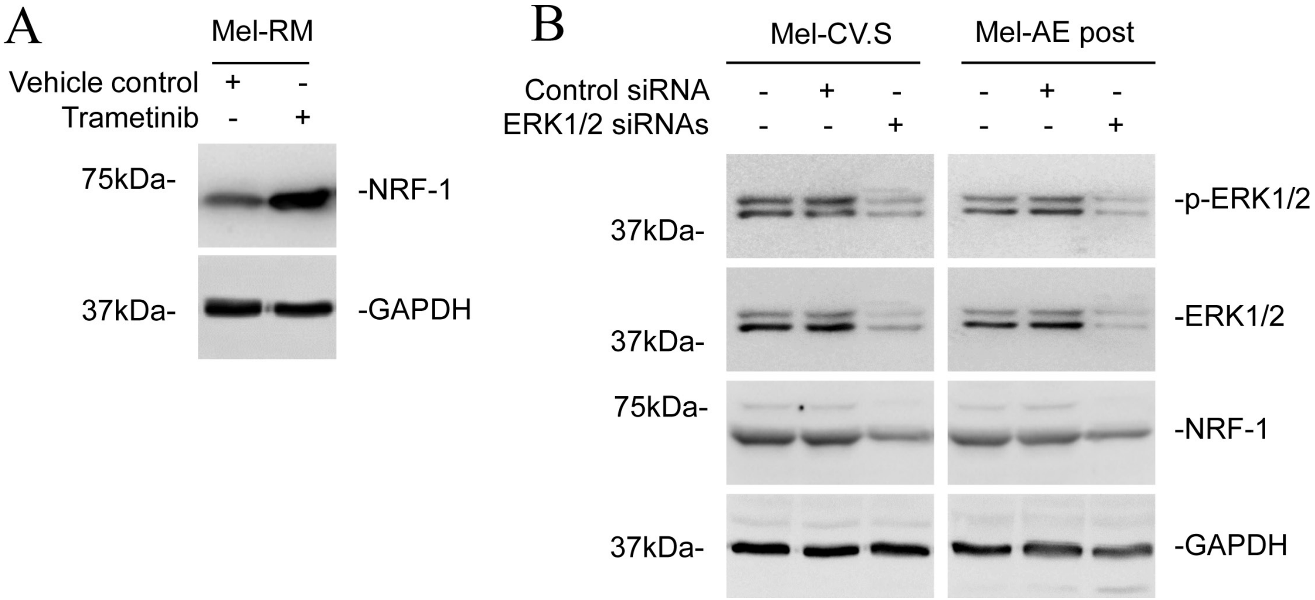

Supplementary Figure 5: (**A**) Whole cell lysates from Mel-RM cells with or without treatment with trametinib (1 μM) were subjected to Western blot analysis. Data shown are representative of three individual experiments. (**B**) Mel-CV.S and post-treatment Mel-AE cells were introduced with the control siRNA or the combination of ERK1 and ERK2 siRNAs. Twenty-four hours later, whole cell lysates were subjected to Western blot analysis. Data shown are representative of three individual experiments.

Supplementary Table 1: List of antibodies

Antibody (Catalogue No.)	Usage	Company
CD47 (B6H12) (sc-12730)	Immunoblotting /Cell surface staining	Santa Cruz Biotechnology (Santa Cruz, CA)
Calreticulin (ab4)	Immunoblotting	Abcam (Melbourne, VIC,Australia)
Calreticulin (ab22683)	Cell surface staining	Abcam (Melbourne, VIC,Australia)
Mouse IgG1, kappa monoclonal [MOPC-21] - isotype control (ab18443)	Cell surface staining	Abcam (Melbourne, VIC,Australia)
NRF-1 (H-300) (sc-33771)	Immunoblotting	Santa Cruz Biotechnology (Santa Cruz, CA)
p-ERK (E-4) (sc-7383)	Immunoblotting	Santa Cruz Biotechnology (Santa Cruz, CA)
GAPDH (6C5) (sc-32233)	Immunoblotting	Santa Cruz Biotechnology (Santa Cruz, CA)
p44/42 MAPK (#9102)	Immunoblotting	Cell Signalling Technology (Beverly, MA)
NRF-1 (ab34682)	ChIP	Abcam (Melbourne, VIC,Australia)
Anti-CD47 [B6H12.2] (ab3283)	Phagocytosis assay	Abcam (Melbourne, VIC,Australia)
Mouse IgG1 [B11/6] - Isotype Control (ab91353)	Phagocytosis assay /Cell surface staining	Abcam (Melbourne, VIC,Australia)

Supplementary Table 2: List of reagents

Reagent	Company
Vemurafenib (S1267)	Selleckchem (Redfern, NSW, Australia)
Trametinib (S2673)	Selleckchem (Redfern, NSW, Australia)
SCH772984 (S7101)	Selleckchem (Redfern, NSW, Australia)
Actinomycin D (A9415)	Sigma-Aldrich (Castle Hill, NSW, Australia)

Supplementary Table 3: List of siRNAs[a]

siRNA	Sense	Anti-sense
ERK1 siRNA	GCUGAACUCCAAGGGCUAUTT	AUAGCCCUUGGAGUUCAGCTT
ERK2 siRNA	GUGCUCUGCUUAUGAUAAUTT	AUUAUCAUAAGCAGAGCACTT
NRF-1 siRNA-1	GGAGGUUAACUCAGAACUG	CAGUUCUGAGUUAACCUCC
NRF-1 siRNA-2	CGUUAGAUGAAUAUACUAC	GUAGUAUAUUCAUCUAACG
Sp1 siRNA	CUAUGAACUACAGGUGUUU	AAACACCUGUAGUUCAUAG
Control siRNA	UUCUCCGAACGUGUCACGUTT	ACGUGACACGUUCGGAGAATT

[a]The siRNAs used were synthesized by GenePharma (Shanghai GenePharma Co, Ltd, Shanghai, China).
